# Ethiopia’s Antibiotic Footprint: Employing the Newly Emerging Digital Concept to Estimate Annual Consumption for the Country

**DOI:** 10.7759/cureus.36013

**Published:** 2023-03-11

**Authors:** Girma Gutema

**Affiliations:** 1 Pharmacology, Rift Valley University, Adama, ETH

**Keywords:** digital health technology, public health policy, pharmaco-epidemiology, antibiotic consumption, antibiotic footprint

## Abstract

Background

The processes involving resistance development against antibiotics have historically been part of the Darwinian evolution. However, the increasing use of antibiotics in modern medicine has intensified the selection pressures with an acute gear-up, rather than as part of this very slow evolutionary process that selects for enhanced fitness for survival. Two major recommendations have been made in the past to tackle this challenge: (1) incentivizing the pharmaceutical industry to invest more in research and development endeavors so that they come up with new antibiotics, and (2) implementing antimicrobial stewardship programs in healthcare systems.

Methodology

In this study, the third and emerging approach, namely, documenting antibiotic footprint, was employed as a communication tool that targets individual consumers of antibiotics. Data obtained from the Ethiopian Pharmaceutical Supply Agency were curated to systematically compile antibiotic consumption at each of the agency’s regional hubs. The exact geospatial locations of the hubs were generated and synchronized to depict the size of the antibiotic footprint infograph as proportional to the antibiotic consumption data at each hub. Moreover, the cumulative and per-capita consumption of these antibiotics at the country level (overall antibiotic footprint) were calculated by including estimated data for the livestock sector.

Results

A total of 698.2 tons of antibiotics were used in Ethiopia in 2018, and the per-capita consumption of antibiotics was 5.8 g per person. Extended-spectrum (J01CA) and beta-lactamase-resistant penicillins (J01CF) were the most commonly utilized classes of antibiotics which accounted for, respectively, 38.3% and 20.8% of all antibiotics used in the country’s public health sector. Hubs in Addis Ababa (14%) and Hawassa (12%) topped the overall antibiotic consumption in the country. Contrarily, hubs in Gambella and Semera received relatively smaller quantities of antibiotics, with totals of 4.8 tons (0.9%) and 10.2 tons (1.9%), respectively.

Conclusions

This study shows that the newly emerging concept of the antibiotic footprint is a simple and suitable tool for public health policy communications targeting individual consumers of antibiotics. If implemented judicially, the concept of the antibiotic footprint has a huge potential to support global scientific efforts and collaborations in setting standards that help to reduce the overuse and misuse of antibiotics in the future.

## Introduction

Historical records on man’s understanding of infectious diseases and their causative agents are centuries old. In the 14th century for instance, two notable Persian scholars, Ibn Khatima and Ibn-al-Khotib, proposed that infectious diseases were caused by what they called “contagious entities” [1], but without definitively describing what those “contagious entities” were. Moreover, using anti-infective agents obtained from natural products is widely ingrained in the practices of traditional medicine in many ancient cultural societies. For instance, analytical investigations employing mass spectroscopic techniques in recent times obtained evidence from human skeletal remains in ancient Nubian civilization (dating back to 350-550 AD) which revealed that the antimicrobial tetracycline extracted from *Streptomyces *bacteria in beer fermentation was used for the treatment of infectious diseases [2].

Fast forward, particularly since the period commonly called “the scientific revolution” (ca 1500-1800) marked by the emergence of modern science, sustained efforts had been underway to discover cures for infectious diseases. Several chemical entities were synthesized in laboratories as part of such scientific endeavors. The major limitations of the chemical entities synthesized in laboratories had not, however, been efficacy, but safety. For example, Salvarsan, an anti-syphilis agent synthesized in 1910 by Ehrlich and, in fact, the first antibiotic ever made by man in a laboratory was too toxic to make it to the clinical catalog. Penicillin was the first safe and efficacious antibiotic discovered in the laboratory by the Scottish scientist Alexander Fleming and his co-workers in 1928 and came to clinical use during the Second World War [3] and in fact because of it.

The series of outbreaks of infectious diseases in Europe during and right after the end of the Second World War, the primary victims of which were wounded soldiers during the war, further boosted the scientific endeavors made to synthesize more effective antibiotics in laboratories. In fact, all of the scientific investigations that ultimately brought penicillin, hitherto forgotten on the Petri dishes of Alexander Fleming’s lab for over a decade, were centrally coordinated and funded by government research agencies and scientific wings of army departments which were in desperate need for anti-infectives for soldiers who sustained wounds in the war [4]. Over the two decades that followed, such momenta were kept as pharmaceutical companies and governments in Europe, Japan, and North America continued funding scientific research. As a result, new classes of antimicrobial agents developed one after another, leading to what is now commonly called the “golden age” of antimicrobial chemotherapy [5,6].

Over the last seven decades, antimicrobial chemotherapy constituted man’s effective strategy to fight infectious diseases with remarkable gains that significantly improved life expectancies in different parts of the world [7]. A report published by the Microbiology Society indicated that between 1944 and 1972, human life expectancy globally increased by an average of eight years, a significant increase primarily attributed to the introduction of antibiotics into clinical care [8].

However, evolution is challenging these gains of the antibiotic revolution in modern medicine. The processes involving resistance development against antibiotics have historically been part of the Darwinian evolution. However, in the backdrop of this evolutionary reality, the ever-increasing use of antibiotics in modern medicine, particularly in the post-penicillin era, has intensified the selection pressures with an acute gear-up rather than as part of the slow and natural evolutionary process that selects for enhanced fitness for survival [9-11].

Two major recommendations were made in the past to tackle this challenge. One is incentivizing the pharmaceutical industry to invest more in research and development endeavors so that they come up with new and novel antibiotics [12,13]. The other is the idea of implementing antimicrobial stewardship strategies in healthcare systems [14,15]. The former targets the pharmaceutical industry to revitalize efforts to bring novel antibiotics to the market, while the latter mainly targets healthcare authorities, health systems management, and administration. And for the latter, different tools to undertake regular surveillance and monitoring of the utilization of antibiotics have been developed to inform relevant policy interventions.

Moreover, these two recommended strategies are absolutely important. However, as much important is also the third one, the active engagement of individual consumers of antibiotics in society through organized campaigns in tackling the problems of misuse and overuse of antibiotics. And for such public awareness campaigns to become effective, generic measurements for antibiotic consumption as well as simple communication tools are key. Currently, available measurement systems for quantifying consumption of antibiotics such as defined daily doses (DDDs), or its derivative DDD per a thousand inhabitants per day (DID), are meant for healthcare professionals and they are technically too complex to understand for nonprofessionals and to the wider public at large.

With this understanding of the existing gap in effectively engaging individual consumers in public campaigns aiming to reduce the misuse and overuse of antibiotics in the community, the concept of the “antibiotic footprint” was recently recommended by researchers as a global communication tool [16,17].

The concept envisages to stimulating behavioral changes practically needed to reduce individuals’ direct and indirect consumption of antibiotics in communities. Adopted from the already established innovative concept of “carbon footprint” and aspiring to build on its successes, antibiotic footprint quantifies the consumption of antibiotics in human health, in animals/livestock, and in agriculture/aquaculture as measured in simpler and generic terms such as the total mass of antibiotics used or per-capita of a population. As carbon footprint captures the mass of greenhouse gases such as carbon dioxide emitted to the environment by every individual, antibiotic footprint quantifies the mass of antibiotics consumed by each individual in the population.

The goal of carbon footprint is to reduce the use of energy generated from fossil fuels and hence emissions of greenhouse gases to a minimum [18]. Likewise, the goal of the antibiotic footprint is to reduce antibiotic consumption to a minimum by cutting their unnecessary use [17]. The underlying idea here is that we have to use energy to live but using too much energy obtained from fossil fuels is driving climate change which has become an existential threat to human survival on planet earth. Likewise, we have to use antibiotics to fight infectious diseases in humans and animals, but overuse and misuse of antibiotics are promoting resistance and increasing the number of people and animals dying due to infections caused by drug-resistant strains of pathogenic microbes.

This study, which was previously posted on Research Square pre-print server on October 24, 2022, aims to employ this newly emerging concept of antibiotic footprint to estimate the gross and per-capita consumption of antibiotics in Ethiopia for the year 2018.

## Materials and methods

Disaggregated data depicting the utilization of antibiotics at all the regional hubs of Ethiopia’s public health sector pharmaceutical supply chain monopoly, the Ethiopian Pharmaceutical Supply Agency (EPSA), for the year 2018 were used in this study.

Data obtained from the central EPSA’s excel spreadsheets on demand quantification and delivery of all medicines, medical equipment, healthcare technologies, medical supplies, and laboratory reagents, all of which constitute “Pharmaceuticals” as per the Ethiopian law, to the regional hubs were curated to systematically compile the consumption of all antibiotics.

These compiled data were used to compute antibiotic consumption at each of the 17 regional hubs of EPSA in human health. QGIS, version 3.4.4, Madeira 2018 (QGIS Development Team, Open-Source Geospatial Foundation Project) was employed to generate the exact geospatial locations of the hubs, synchronized to depict the size of the antibiotic footprint infograph as proportional to the antibiotic consumption data at each hub. The percentages of different classes of antibiotics, at the fourth level of the ATC classification index [19], consumed in 2018 were also calculated. Moreover, the cumulative and per-capita consumption of these antibiotics at the country level (antibiotic footprint) were calculated by including data from the livestock sector. Due to a lack of official data available on the consumption of antibiotics in the livestock sector in Ethiopia, estimate figures offered by Precise Consult International [20] were used. Official population census data for Ethiopia are not available for the year 2018 and, hence, data from the World Bank’s population projections portal [21] were used in computing per-capita antibiotic consumption.

Moreover, percentage expenditures on all antibiotics were calculated from EPSA’s registers compared to that of the total expenditures on all medications. Results are presented as text, figures, and infographics.

## Results

Per-capita consumption of antibiotics for Ethiopia in 2018 was 5.8 g per person. In the public sector, which accounts for about three-quarters of healthcare service delivery, percentage expenditure on antibiotics (considering procurement prices at state-owned pharmaceutical supply chain monopoly, EPSA) accounted for 37.3% of the expenditures on all medications.

**Figure 1:**
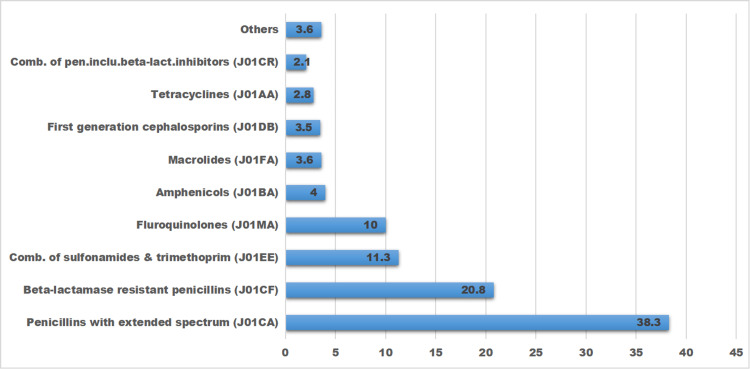
Percentages of antibiotic classes delivered to regional hubs based on the quantification of their annual demands for the year 2018.

Penicillins with extended-spectrum (J01CA) and beta-lactamase-resistant penicillins (J01CF) were the most commonly utilized classes of antibiotics in 2018 which accounted for, respectively, 38.3% and 20.8% of all antibiotics used in the country’s public health sector in 2018 (see Figure 1).

**Figure 2:**
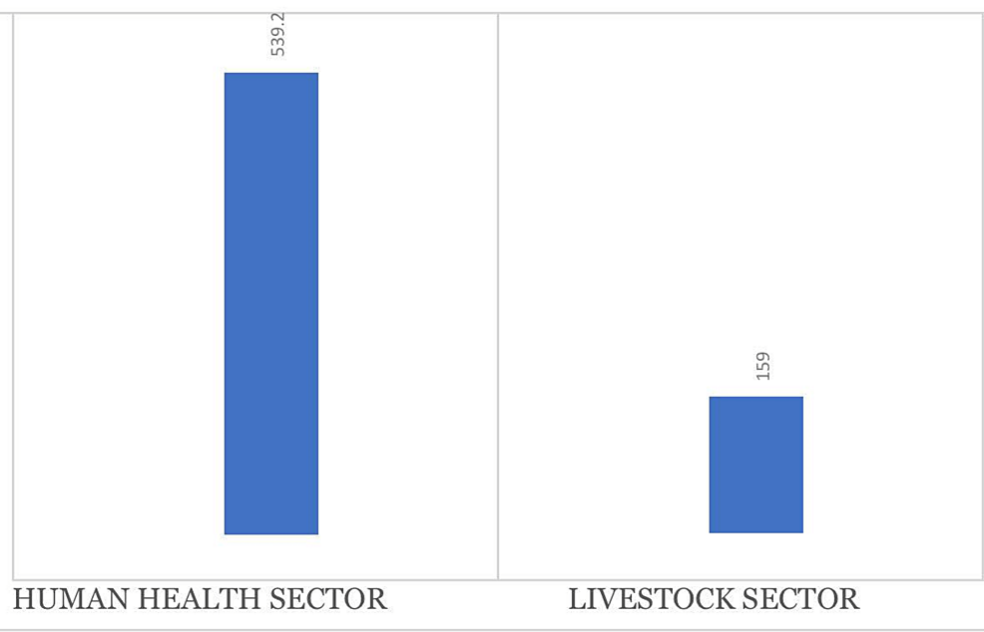
Annual consumption of antibiotics (in tons) in the human health and livestock sectors in Ethiopia in 2018

In 2018, a total of 698.2 tons of antibiotics were used in Ethiopia, of which about three-quarters accounted for use in the human healthcare sector (Figure 2).

**Figure 3:**
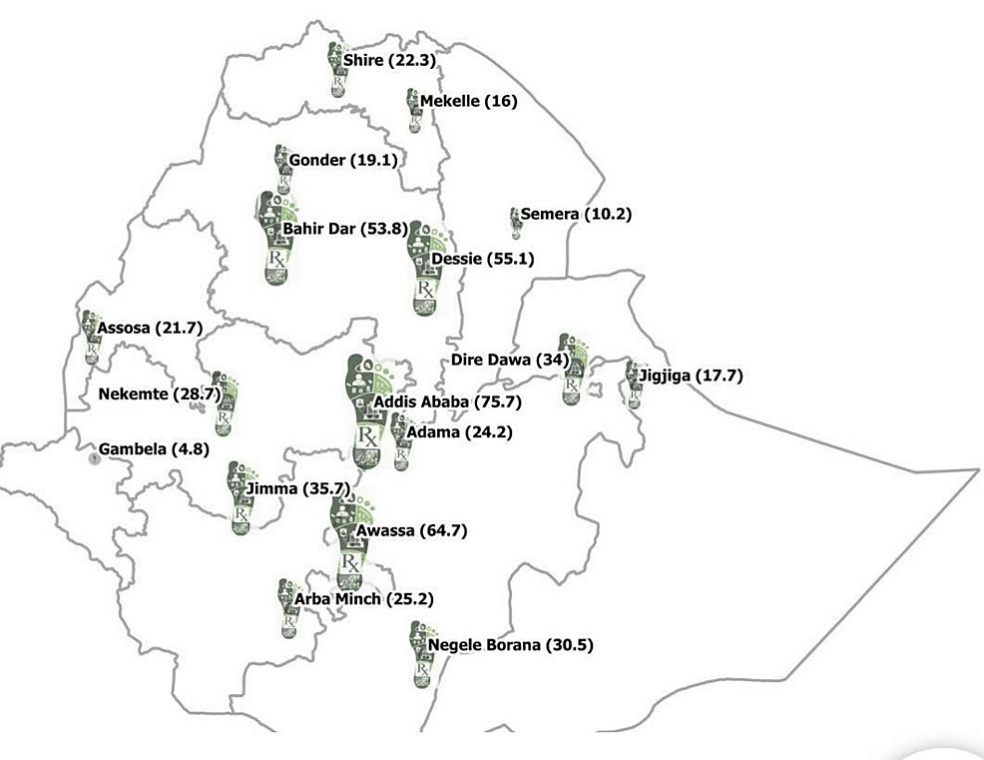
Antibiotic footprint as quantities of antibiotics (in tons) consumed at EPSA’s regional hubs in Ethiopia in 2018. Source: own design based on collected data. EPSA = Ethiopian Pharmaceutical Supply Agency

When the antibiotic consumption data in human health were disaggregated into the 17 regional branches that constituted the EPSA’s supply chain hubs in the study year, the hubs in Addis Ababa (14%) and Hawassa (12%) topped in terms of the quantities of antibiotics they received. Contrarily, hubs in Gambella and Semera received relatively smaller quantities of antibiotics, with totals of 4.8 tons (0.9%) and 10.2 tons (1.9%), respectively (Figure 3).

## Discussion

**Figure 4:**
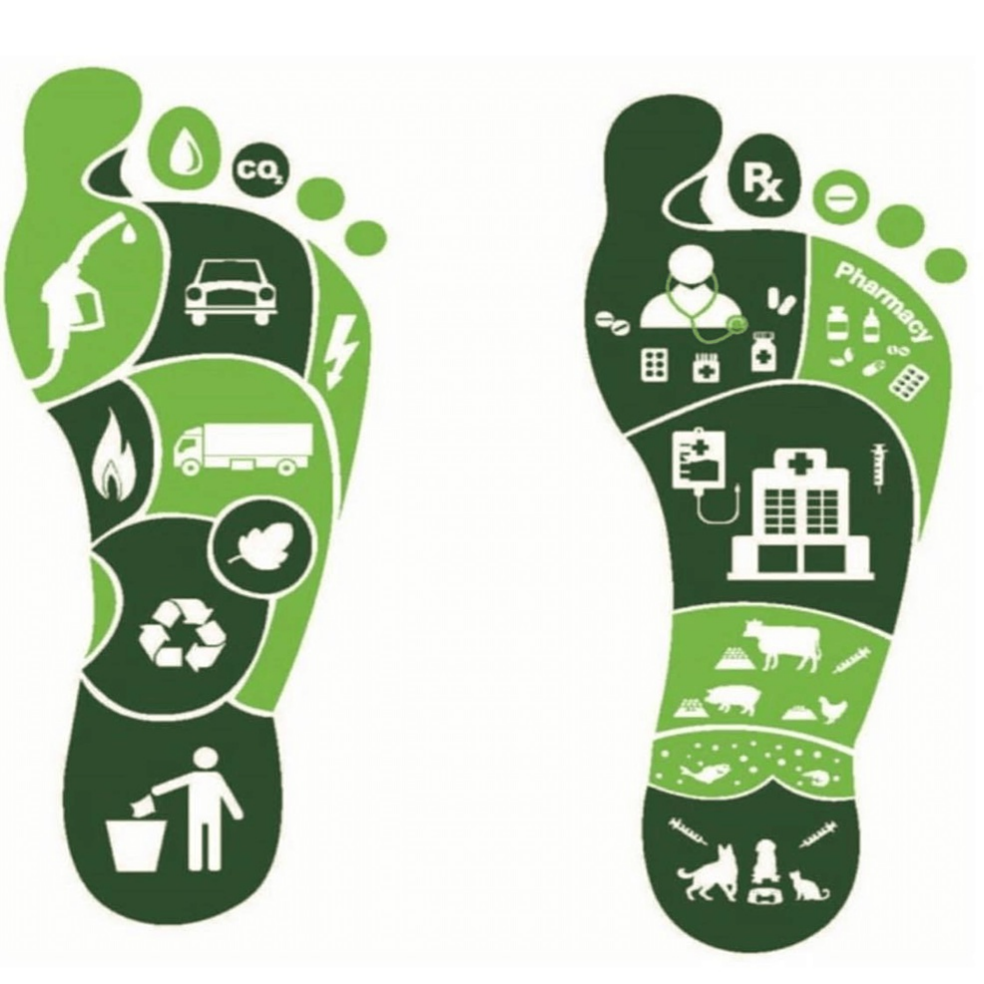
A comparative conceptual figure for carbon footprint and antibiotic footprint, showing holistic approaches to the former (left) and the latter (right), respectively. Source: AntibioticFootprint.net, CCBY 4.0 [17].

In this study, the newly emerging digital concept of the antibiotic footprint was employed to estimate the cumulative and per-capita consumption of antibiotics in Ethiopia for the year 2018, which is a holistic estimate for the human and livestock health sectors. The concept is termed digital because, like carbon footprint, it is powered by a digitized and generic platform on the world wide web. Consumers are no doubt in a strong position to influence antibiotic misuse and overuse. The concept, therefore, envisages to reinvigorating the role of individual consumers of antibiotics in society in tackling the challenges of antibiotic resistance by stimulating positive behavioral changes needed to reduce the overuse and misuse of antibiotics. This concept is gaining traction among policymakers worldwide, much like the hitherto established concept of the carbon footprint from which it was adopted, and an online interactive database pulling official data on antibiotic consumption in human health, livestock, and agricultural sectors from various countries have been established. The online interactive database for antibiotic footprint was launched quite recently, but it has already stored data since the year 2010 for many countries. Figure 4 below shows a comparative conceptual picture of carbon footprint and antibiotic footprint [17].

The concept of antibiotic footprint also complements the other newly emerging concept called the “One Health Concept” [22] because it measures the cumulative consumption of antibiotics in the human and animal health sectors. As such, it captures both the direct and indirect consumption of antibiotics by the community within the wider ecosystem. Most antibiotics used in the animal health sector are often excreted intact into the environment and, subsequently, play part in adding selective pressure on the development of resistance against antibiotics within the wider ecosystem, and, hence, the rationale for including antibiotics consumed in the animal healthcare sector in computing the antibiotic footprint [16].

Comparing the findings of the current study with antibiotic footprints for two African countries (Sudan and Tanzania) whose official data have been published in the online interactive database [17] may help fine-tune and capture the practical underpinnings of the concept. Accordingly, the gross annual consumption of antibiotics in the human health sector for which the latest data are available for these African countries (2015 for Sudan and 2016 for Tanzania) are quite comparable with the findings in the current study: 675.8 tons, 698.2 tons, and 712.5 tons for Sudan, Ethiopia, and Tanzania, respectively. However, if adjustments are made for differences in total populations between the countries and the estimated proportion of antibiotic consumption in Ethiopia’s private healthcare sector is factored in, the resulting per-capita figures show that a Sudanese citizen consumed about twice more antibiotics than an Ethiopian, while an Ethiopian consumed three-quarters of the antibiotics consumed by a Tanzanian. The assumption underlying the calculations in these comparisons here was, of course, that there existed no significant increase in the annual consumption of antibiotics between 2015-2018 and 2016-2018 for Sudan and Tanzania, respectively.

Nevertheless, differences in the antibiotic footprints among countries could be attributed to various factors such as healthcare-seeking behaviors of the population, socioeconomic differences, variations in the epidemiological distributions of infectious diseases, and differences in agricultural practices in different countries, among others. Furthermore, importantly, comparison of such data can influence peoples’ curiosity, thereby helping bring practical behavioral changes which can positively contribute to efforts made to reduce individual antibiotic consumption such as keeping environmental sanitation, taking vaccinations, keeping personal hygiene, considering food choices, and avoiding self-medication practices or over the counter use of antibiotics [16].

Ethiopia is a very vast country with multiple physical features of geography and ecological systems ranging from arid lowland areas to scorching-sun deserts, to tropical highland plateaus, and all the way to wet mountainous hinterlands. Such ecological variants are obviously important determinants for the differential distribution of diseases, and, hence, the use of a longer list of antibiotics and other medications in the country seems to be a logical consequence of that. Particularly outstanding data in this study was that cloxacillin was the sole antibiotic product that did exclusively account for the level of consumption (20.8%) constituted by beta-lactamase-resistant penicillin (J01CF) whose total consumption came second only to extended-spectrum penicillin (J01CA). On the other hand, ampicillin and amoxicillin were the two products added up to constitute 38.3% of consumption accounted by extended-spectrum penicillin (J01CA).

A countrywide comprehensive study on the prevalence of resistance against antibiotics has yet to be undertaken in Ethiopia, but a few studies conducted at the level of healthcare institutions reported a high percentage of resistance against some of these commonly used antibiotics as ampicillin and amoxicillin [23,24]. The encouraging development on this, however, is that the country has recently established an Antimicrobial Resistance Surveillance system that works through laboratory-based sentinel sites in healthcare institutions [25]. Operational since July 2017, it envisages pulling data from the sentinel sites to centrally monitor patterns of antimicrobial resistance in the country. This sentinel site-based antimicrobial resistance surveillance system, which is being coordinated under the auspices of the Ethiopian Public Health Institute, released its first report in August 2018 [26], which essentially corroborated the findings of the earlier health facility-based studies that reported high percentages of resistance against such commonly used antibiotics, as documented in this study, as ampicillin, amoxicillin, ciprofloxacin, and cotrimoxazole, among others. Comparably, high consumption of extended-spectrum penicillin (J01CA) and beta-lactamase-resistant penicillin (J01CF) were reported in our earlier health facility-based study [27] as well as elsewhere [28].

**Figure 5:**
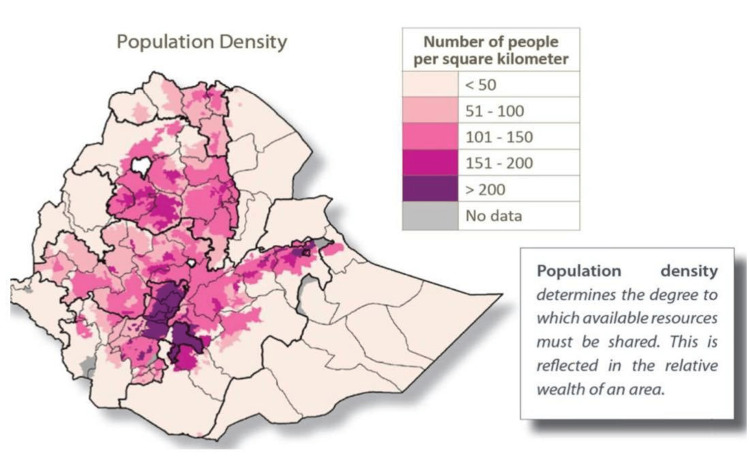
Population settlement density in Ethiopia. Source: Center for Rural Development (SLE) Berlin, Discussion Paper 03/2017, p.6. The discussion paper is available online as open-access material [29].

Moreover, the distributions of disaggregated data for antibiotic consumption at Ethiopia’s regional hubs (for human health) appear to draw parallels with the patterns of population settlement density in Ethiopia, and, hence, the subsequent pressure on the demands for healthcare service provisions. See Figure 5 [29] and compare the patterns with the regionally tailored antibiotic footprint shown in Figure 3 (note that the sizes of the infographic footprint shown in Figure 3 correspond to the total consumption of antibiotics at the regional hubs).

This is to say, hubs situated in densely populated parts of the country and hence serve healthcare facilities within such catchment areas such as in central (e.g., Addis Ababa, Adama), south/south-west (e.g., Hawassa, Arba Minch, Jimma), north/north-west (e.g., Dessie, Bahir Dar), and in eastern parts (e.g., Dire Dawa) have a relatively higher consumption of antibiotics. On the contrary, lowland areas where we have healthcare facilities that serve sparsely populated parts of the country such as in the western lowland frontiers (e.g., Gambella) and eastern lowland areas (e.g., Semera, Jigjiga) got relatively lower consumption of antibiotics. Antibiotic consumption in the remaining hubs falls far in between, and that too corresponds well with the population density within the catchment areas of their services. In addition to that, differences in the epidemiological distribution of infectious diseases, determined by the ecological variations as discussed above, must have also played their part.

The strength of this study is that it employed the newly emerging digital concept of antibiotic footprint to estimate the total, as well as the per-capita annual consumption of antibiotics in Ethiopia for the year 2018. The study can, therefore, offer important and countrywide refined input of datasets to the initiative now underway in Ethiopia to institutionalize the antimicrobial stewardship system through the sentinel-based resistance surveillance program established in 2017.

The study is not, however, without its limitations. First, the data for the consumption of antibiotics in the livestock sector used in the current study are not primary data as were the case for those in the human health sector, which is due to the lack of complete and compiled data availability for the former in Ethiopia’s official registers. For the data in the livestock sector, we had to rely on documented scientific projections, i.e., secondary data, offered by expert consultants at Precise Consult International in which they estimated the annual consumption of antibiotics in Ethiopia for the year 2018 based on the 2012 data. This means that the quality of our data on antibiotic consumption for the livestock sector are only as good as secondary data could be. Second, our data for annual antibiotic consumption in the human health sector did not include antibiotics used within the private health sector in Ethiopia due to a lack of complete and compiled official data. Official reports show that the private health sector in Ethiopia accounts for some 27% of the total number of healthcare facilities available in the country [30]. Hence, if we assume that the sector could possibly account for the same proportion of the total annual consumption of antibiotics in the country, this might have had an effect of a downward bias (to that extent) on our statistical estimate of Ethiopia’s antibiotic footprint for the year 2018.

## Conclusions

This study shows that the newly emerging concept of the antibiotic footprint is a simple and suitable tool for public health policy communications targeting individual consumers of antibiotics in the community. The data and our interpretation of them in this study suggest that if implemented judicially, the digital concept of antibiotic footprint has a huge potential to support global scientific efforts and collaborations in setting standards that help to reduce the overuse and misuse of antibiotics in the future.
